# Uniaxial 3D Measurement with Auto-Synchronous Phase-Shifting and Defocusing Based on a Tilted Grating

**DOI:** 10.3390/s21113730

**Published:** 2021-05-27

**Authors:** Hui Ren, Yuankun Liu, Yajun Wang, Ningyi Liu, Xin Yu, Xianyu Su

**Affiliations:** Department of Opto-Electronic, Sichuan University, Chengdu 610065, China; renhui@stu.scu.edu.cn (H.R.); yjwangisu@scu.edu.cn (Y.W.); 2017222050010@stu.scu.edu.cn (N.L.); 2018222050049@stu.scu.edu.cn (X.Y.); xysu@scu.edu.cn (X.S.)

**Keywords:** uniaxial, techniques, auto-synchronous phase-shifting, tilted focused image plane (FIP), tilt focusing, rotate scanning

## Abstract

Conventional uniaxial techniques generally require shifting objects or projection grating with the assistance of a high-precision mechanical moving component. To overcome this limitation, we propose a novel uniaxial 3-D shape measurement system with auto-synchronous phase-shifting and defocusing by using a tilted and fixed projection grating. The tilted focused image plane (FIP), which is reflected by a mirror at about 90 degrees, could be shifted across the measured surface by slightly rotating the mirror within a small angle range. This procedure will simultaneously introduce the change in defocusing and phase-shifting of the fringe. The modulation curve of each point can be deciphered by Fourier fringe analysis after a sequence of fringe intensities is acquired. Since both the measured object and projection grating are fixed, the proposed method could make the measurement system more compact and flexible. Both computer simulation and experiments are carried out to demonstrate the validity of this proposed system.

## 1. Introduction

The uniaxial technique, which is also called the vertical measurement technique, can overcome the inherent shadow and shutoff drawback of the triangulation by the coaxial optical system for projection and observation [1]. In the uniaxial measurement methods, the surface height information of the tested object is encoded into the defocusing degree of the images [2]. Therefore, it aims at measuring those objects with sharp changes, such as deep holes and stairs.

The quantitative relationship between the height and defocusing degree may be based on contrast [3], phase error [4] and modulation [5]. When focusing on the configurations of the Modulation Measurement Profilometry (MMP) systems, the methods could be divided into roughly three types according to the setups. The first category is to fix the system and shift the tested object. It can easily decipher the height because the scanning step is the sampling height step as well. However, it only works well with the telecentric system [3,6]. Yang et al. [6] built a structured illumination microscopy system (SIM) to acquire the height from the deciphered contrast curve by Fourier transformation technique. The key of the kinetics is to scan the object along the z-direction with a high-accuracy translation stage and the synchronous digital phase-shifting. The second one is to fix the object and the imaging system, only shifting the projection part of the whole measuring system. Su et al. [5] proposed a method to shift the projector along the projection axis and calculate the modulation of all the pixels on the object with phase-shifting. Y. Mizutani et al. [7] used a varifocus lens, whose focus length can be continuously varied from a concave to a convex shape by changing the liquid pressure. Jing et al. [8] combined an EF projection lens and DMD grating to realize a high flexible focus change process, which is also used in the recent work [9].

The third category avoids any scanning process by fixing the object as well as the whole system. Dou et al. [10] employed two separated orthogonal gratings combined by a beam splitter to form two different focused image planes. Then, a modulation ratio would be obtained from the two modulations, which were extracted from a single-shot captured image by Fourier fringe analysis [11]. Later, Lu et al. [12] used a combined orthogonal grating and produced two different focused image planes by a cylinder lens. Xu et al. [4] used phase error as a quantitative criterion to build the implicit relationship between the height and the defocusing degree. Here, the phase error was calculated by the phase obtained from the binary defocused pattern and that obtained from the ideal sinusoidal patterns. Later, Liu et al. [13] obtained the phase error by only using the binary fringe patterns. The improvement included using a large-step phase-shifting technique to obtain the ideal phase, and the four-step phase-shifting technique to keep the third harmonic instead of the three-step one to produce a much larger nonlinear error. In a word, the existing methods are normally based on the defocusing curve along the optical axis, i.e., for a single object point, its intensity curve is based on the intensities changed along the optical axis. However, the curve can also be stemmed from the neighbor pixels of the projected fringe. Then, it is possible to produce a measuring system without the high-precision scanning mechanism along the z-direction.

In this paper, a novel MMP method with auto-synchronous phase-shifting based on tilted grating and rotating scanning is proposed. The grating, which is set at a certain angle along the optical axis, will produce a tilted fringe image plane. Therefore, a Gaussian modulated fringe image will be casted on a certain reference plane, which is perpendicular to the projecting axis. Meanwhile, a mirror is introduced to rotate the projecting axis about 90 degrees. Then, by rotating the mirror, it will produce two shifts, one is to shift the projected fringe image laterally, the other is to shift the peak of the Gaussian modulated fringe image. Then, a modulation curve for each pixel could be obtained from a sequence of the intensity values by Fourier transform technique. The height could be reconstructed from the calibrated mapping parameters after obtaining the location of the modulation peak. Computer simulation and experiments verify its effectiveness and feasibility.

## 2. Principle

### 2.1. The Structure of the Proposed Measurement System

**Figure 1:**
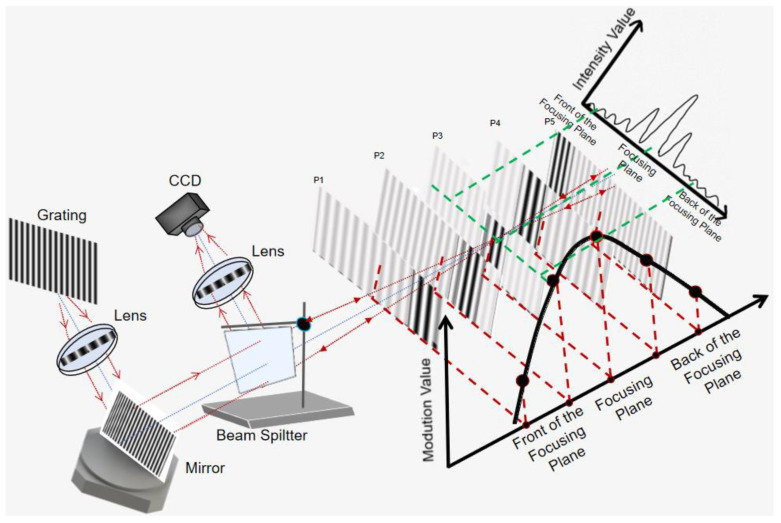
The schematic of the proposed MMP.

We proposed a novel uniaxial 3-D shape measurement system by using tilt focusing and rotate scanning, with the schematic drawing of the MMP shown in Figure 1.

The sinusoidal grating, which is tilted at a certain angle of α along the projecting optical axis instead of the classical perpendicular setting, will produce a tilted FIP of the fringe image in the whole measuring volume. A mirror, which is placed after the projection lens, will reflect the projected fringe image by about 90 degrees. For the simplicity, we assume the grating line is perpendicular to the optical axis. Then, considering a reference plane, which is placed perpendicular to the optical axis like that of the classical MMP, it will collect a partially focused image. The intensity value can be regarded as following Gaussian distribution shown in upper right corner.

### 2.2. The Measuring Principle of the Proposed Measurement System

**Figure 2:**
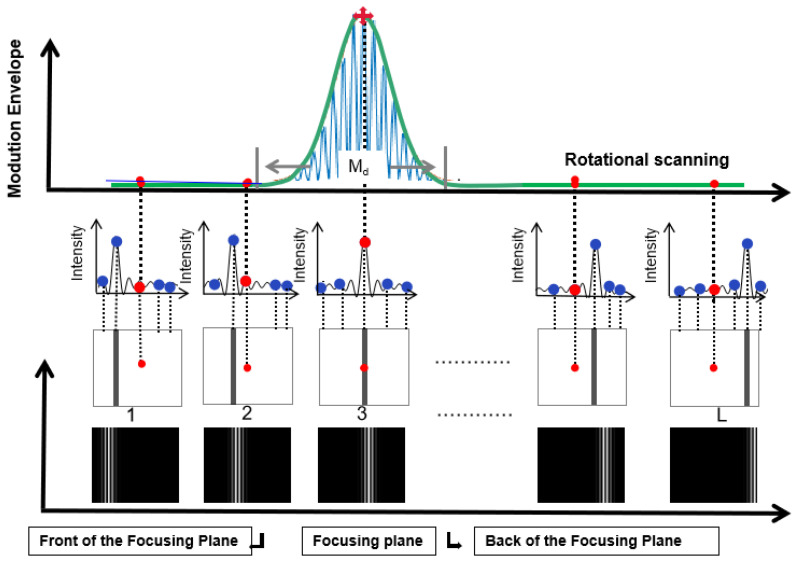
K modulation values for the same pixel.

There is a direct relationship between the focused position of the fringe image and the height of the reference plane, i.e., a different-height reference plane will produce a different focused fringe image. When the reference plane is fixed and we focus on a single point, it is undoubtedly that the FIP could be shifted across the object point by rotating the mirror controlled by the pulse signal with a rotation interval Δ*θ*. When we collect a sequence of the fringe images, we can obtain a series of fringe intensities for the measured object point as shown in Figure 2. Meanwhile, an auto-synchronous phase-shifting will be fulfilled as well. Therefore, only one fringe image is required for a rotation angle value of *θ_k_*.

As shown in Figure 2, taking K frame images as an example, a sequence of fringe intensities, which include the surface information of the object and are displayed in the green dotted frame, are captured by CCD camera via the beam splitter. Then, an intensity curve *I_k_*(*x*,*y*) will be collected by grouping all images from all different images; here, *k* is the serial number of the image. This envelope called the modulation curve, which is shown as a green solid curve, has a direct relationship with the defocusing degree of the fringe image and can be deciphered by Fourier fringe analysis [14]. Here, the rotation of the mirror by a certain interval can be regarded as sampling the intensity curve. Obviously, the peak of this curve is the location of the focused fringe image exactly, i.e., the height of the object can be obtained as long as the location of the peak is detected.

Note that, although the rotation will slightly damage the uniaxial condition, it will not bring significant occlusion or shadow because the rotation range is only several degrees. Even though every point will produce a different magnification (*M*) and depth of focus (DOF) theoretically, it can be approximated as a constant as well while there are only some fringes in the modulation curve. For an object point, the intensity can be mathematically formulated as

(1) $$I_{k}\left(x,y\right)=\frac{R\left(x,y\right)}{M^{2}(x,y)}\left[A\left(x,y\right)+B\left(x,y\right)\cos\left(\Phi_{0}\left(x,y\right)+2\pi \frac{d\Delta \theta }{p}k\right)\right]$$

where *R*(*x,y*) is the reflectivity of the object, *M*(*x,y*) is the magnification, *A*(*x,y*) is the background intensity, *B*(*x,y*) is the contrast changed by the defocusing of scanning stripes, and Φ_0_(*x,y*) is the initial phase. *d* is approximated as the image distance while the mirror is close to the projection lens. *p* is the period of the fringe casted on the object and its derivative 1/p is the fringe frequency *f*_0_. Δ*θ* is the rotation interval. *k* is the serial number of rotations; its value range is 0~K.

Normally, the point spread function (PSF) is used to represent the defocusing degree [15]. It is modeled as a 2-D Gaussian of the form

(2) $$G(x,y)=\frac{1}{2\pi \sigma^{2}}\exp\left(-\frac{x^{2}+y^{2}}{2\pi \sigma^{2}}\right)$$

where *σ* is the spread parameter, which is proportional to the radius r of the fuzzy spot [15]. Then, the intensity of each point can be written as

(3) $$I_{k}^{’}(x,y;\sigma )=G(x,y;\sigma )*I_{k}\left(x,y\right)$$

where symbol * denotes convolution operation. Then, the defocused image can be expressed as

(4) $$I_{k}^{’}\left(x,y;\sigma \right)=\frac{R\left(x,y\right)}{M^{2}(x,y)}\left\{A\left(x,y\right)+B_{0}\left(x,y\right)\exp\left(-\frac{1}{2}f_{0}^{2}\sigma^{2}\right)\cos\left[\Phi_{0}\left(x,y\right)+2\pi \frac{d\Delta \theta }{p}k\right]\right\}$$

In our novel MMP system, the modulation curve *M_k_*(*x*,*y*), shown as the green solid curve in Figure 2, by applying Fourier transform method [11] from the curve *I^’^_k_*(*x*,*y*) three terms can be obtained and expressed as

(5) $$G_{k}(f)=F[I_{k}^{’}]=G_{0k}(f)+G_{+1k}(f)+G_{-1k}(f)$$

In this formula, *G*_0_*_k_*(*f*) denotes the zero spectrum of the curve, and *G*_+1_*_k_*(*f*) and *G*_−1_*_k_*(*f*) denote the fundamental spectra of the curve. By selecting the appropriate filter window to filter out the useful fundamental frequency component and doing inverse Fourier transform, the modulation envelope *M_k_*(*x*,*y*) can be obtained as

(6) $$M_{k}(x,y)=0.5\frac{R\left(x,y\right)}{M^{2}(x,y)}B(x,y)\exp\left\{-i\left[\Phi_{0}\left(x,y\right)+2\pi \frac{d\Delta \theta }{p}k\right]-\frac{f_{0}^{2}\delta^{2}}{2})\right\}$$

To obtain the height, the peak position of the modulation curve, known as the number of the sequential images, should be extracted. In geometric optics approximation, the modulation distribution before and after the image plane can be approximated as symmetric [16]. The serial number *S_k_*(*x,y*), corresponding to the maximum value of the same pixel during the K scan, can be calculated by the gray gravity center method:

(7) $$S_{k}\left(x,y\right)=\frac{\sum_{k=0}^{K-1}M_{f}^{k}(x,y)\times k}{\sum_{k=0}^{K-1}M_{f}^{k}(x,y)}$$

Note that the peak locations are influenced by both the heights and the lateral positions of the object. For instance, a reference plane, which is perpendicular to the optical axis, can be thought of as at the same height; however, the different points on a row will produce the different serial number. Therefore, a mapping relationship needs to be built in advance. The calibration plane covers the entire measurement range of the system, which is 20 mm in our experiment. The interval between two adjacent planes is 4 mm. Therefore, the reference planes are placed at 0.00, 4.00, 8.00 … 20.00 mm, respectively, by a linear translation stage PI with a 0.001 mm repeated positioning precision. The plane farthest to the beam splitter is set as the base plane, whose height is H(I-1) = 0. The scanning method of the calibration procedure is the same as that of the measurement, as shown in Figure 1. The range of the rotation angle is [−5.5°, 5.5°], and the rotation step Δ*θ* = 0.02°. For each calibration plane, there are 550 frames of the fringe patterns captured by triggering CCD synchronously with the mirror’s rotation. Both during the calibration and measurement process, the start position of rotation is set at the leftmost of the field of view (FOV). For one particular pixel (*x*,*y*), the intensity curve consists of 550 points. Then, a serial number will be obtained for each calibration plane at each camera pixel. The calibrated mapping is represented by quadratic polynomials as

(8) $$H\left(x,y\right)=a\left(x,y\right)+b\left(x,y\right)*S\left(x,y\right)+c\left(x,y\right)*S^{2}\left(x,y\right)$$

*H_i_*(*x,y*) is the actual height value of the i_th_ calibration plane. Each pixel in the images will have its unique formula with different values of quadratic curve fitting coefficients *a*(*x,y*), *b*(*x,y*) and *c*(*x,y*), respectively. So far, the profile of the tested object can be reconstructed according to this coefficients look-up table.

**Figure 3:**
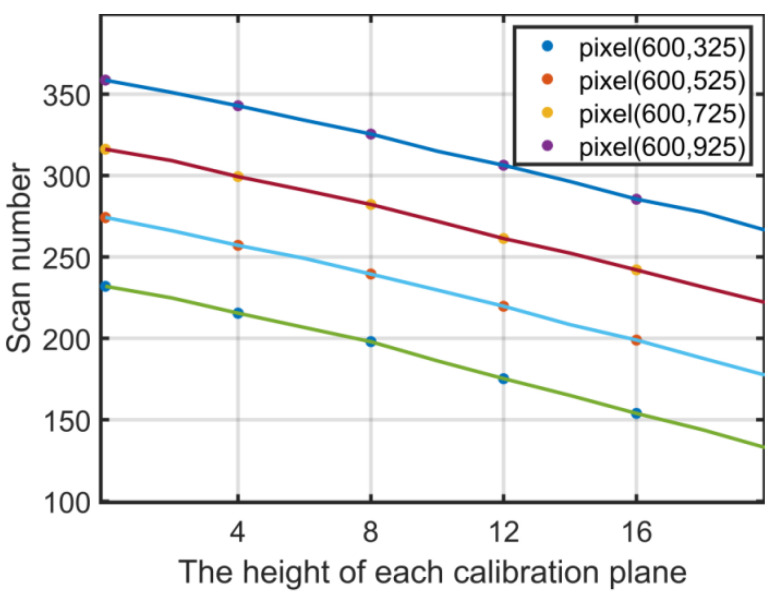
Calibration result of four pixels.

Figure 3 shows the maximum modulation index of the four pixels in each calibration plane used for calibration. The vertical coordinate represents the peak location of the modulation curve, the horizontal coordinate represents the actual physical height of each calibration plane, and the lines represent the interpolation fitting line of the obtained data. Each point in the images will have its unique line, similar to Figure 3.

The depth-of-modulation (*M_d_*), which can directly determine the parameters of the projection system in the measurement system [17], will affect the number and interval of scanning in the measurement process. It is defined as the main lobe width of the front and rear modulation system distribution curve of grating imaging (as shown in Figure 2). In the geometric optical approximation, considering the single lens projection system, the depth-of-modulation of the same pixel on the timeline can be expressed as follows:

(9) $$M_{d}=\frac{2.44d_{0}fMp}{D\left(d_{0}-f\right)}$$

where *d*_0_ is the distance between the grating and the lens, that is, the object distance, *f* is the focal length of the projection lens, and *D* is the aperture of the projection lens. The other two parameters *p* and *M* have the same meaning as Equation (1). Equation (9) can be further simplified as:

(10) $$M_{d}=2.44\frac{M}{D\left(\frac{1}{f}-\frac{1}{d_{0}}\right)}p=Cp$$

where *C* is a constant determined by the projection system parameters. *M_d_*, which acts as the equivalent wavelength in the phase measurement techniques, is exactly equal to the depth range in which the modulation is changed from minimum to maximum to minimum. Generally, the grating is usually placed in front of the projector at a distance of *f*~2*f*. According to Equation (10), after selecting the projector parameters, the smaller the space period of the grating corresponds to the sharper the distribution of the curve and the more sensitive to the depth change. These requirements are handled by thorough design of the *M_d_*. The theoretical accuracy of the measurement system is approximately 1/20 of the *M_d_* of the system, i.e., about 0.2 mm can be measured in this category.

## 3. Simulation

**Figure 4:**
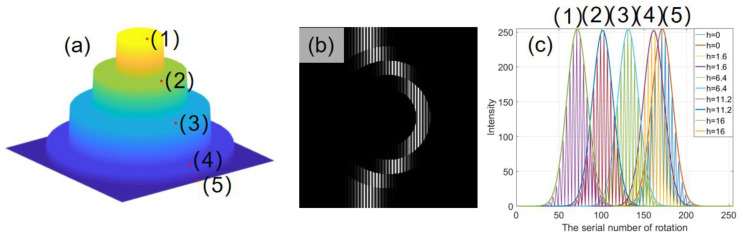
Simulation: (**a**) a step with four discontinuous height and five pixels in the same column; (**b**) the 100th frame of the total 257 frame fringe patterns when the scan step is 2 pixels; (**c**) the intensity curves of five pixels.

In order to verify the proposed method, the tested object simulated with four discontinuous height round steps is shown in Figure 4a and five points at different heights in the same column of CCD array are marked with red dots. The corresponding heights from the bottom to the top are, respectively, 0, 1.6, 6.4, 11.2, and 16 mm. The other parameters in this system are chosen as follows: the period of the projected fringe is 16 pixels, and the size of the fringe patterns captured by CCD camera is 512 × 512 pixels. For simplicity, it is assumed that the shift step of the fringe patterns on the camera caused by rotating is a constant in the whole field of view (FOV). The scanning steps are set to be one, two and four pixels separately to analyze the influence. Apparently, the larger the scanning step, the less images are collected, which will shorten the measuring time and improve the efficiency. To match the experiment, a random noise of 2% fringe intensity is added in the images. The different focal plane positions corresponding to different height positions after each rotation are recorded; a frame of the picture is shown in Figure 4b.

**Figure 5:**
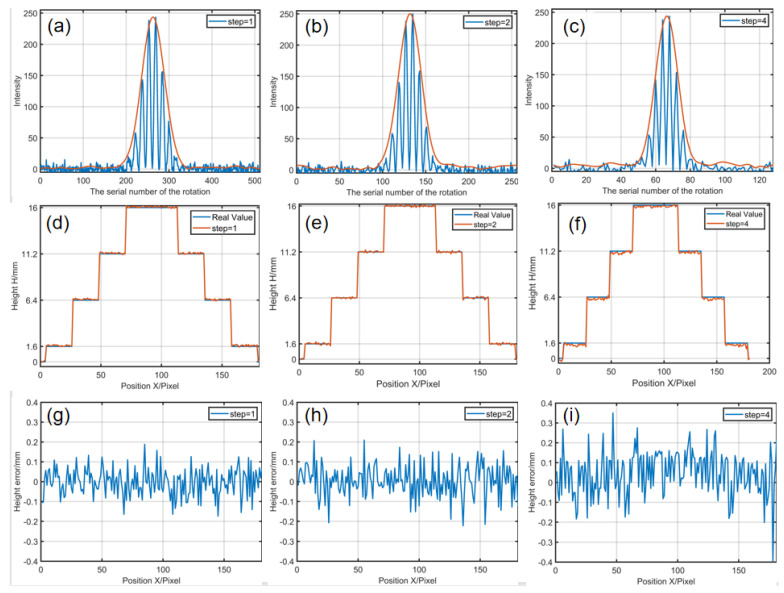
Simulation: (**a**–**c**) the intensity curves and envelopes for different sampling steps; (**d**–**f**) reconstruction cross-sections of the 90th line; (**g**–**i**) the error of reconstruction results.

After scanning procedure, the modulation envelope of eponymous pixels can be obtained by extracting the fundamental frequency component of the intensity information by Fourier transform. Figure 4c shows the fringe amplitudes and envelopes at five different heights, and the 3-D information of the tested object can be obtained according to the maximum sequence number of the modulation system. Figure 5a–c shows the intensity curves and the corresponding envelopes at the height of 11.2 mm with different scanning steps. Note that the modulation envelopes can be considered sampled with different frequency signals. The profiles of the reconstructed 3-D information with different sampling steps are shown in Figure 5d–f. Figure 5g–i show the errors. The standard deviation (STD) errors are 0.0686, 0.0800 and 0.1109 mm, respectively. It is clear that the smaller sampling step will produce a better result at the same noise level. However, it needs more frames.

## 4. Experiment

To further evaluate the performance of the proposed method for measuring complex objects, we carried out experiments to measure a tooth-model using the proposed scheme.

**Figure 6:**
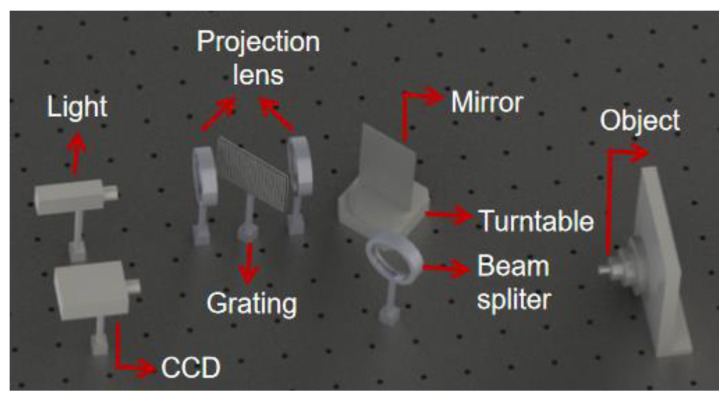
Measurement system device diagram.

This measuring system as shown in the Figure 6, includes a CCD camera (IDS UI-2250SE). Its resolution is 1600 × 1200 pixels and the lens assembled to the camera has a focal length of 50 mm. Grating pitch is 0.67 mm. The range of the rotation angle and the rotation step are the same as that of the system calibration. The rotation accuracy of the turntable (GCD0401-2100M) is 0.001°. A total of 550 images are captured.

**Figure 7:**
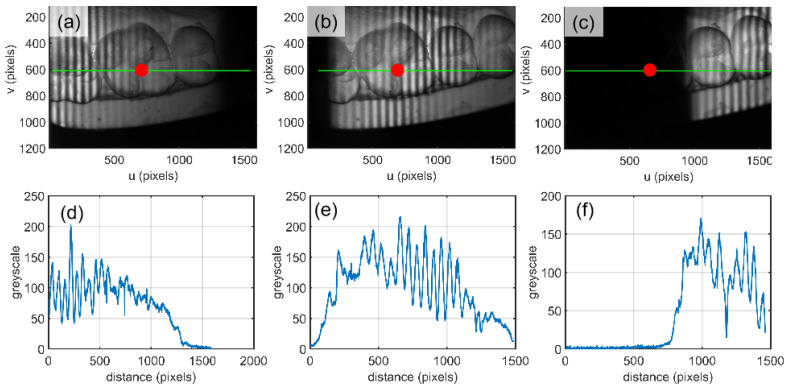
Original intensity map for the measurement of a tooth model: (**a**–**c**) three images at S(i) = 150, 300 and 450; (**d**–**f**) the intensity curves of the same row marked by green lines from three images in (**a**–**c**).

Figure 7a–c shows how the grating pattern was projected onto the tooth-model and the position of FIP was changed with the mirror’s rotation. Figure 7d–f shows the corresponding intensity changes.

**Figure 8:**
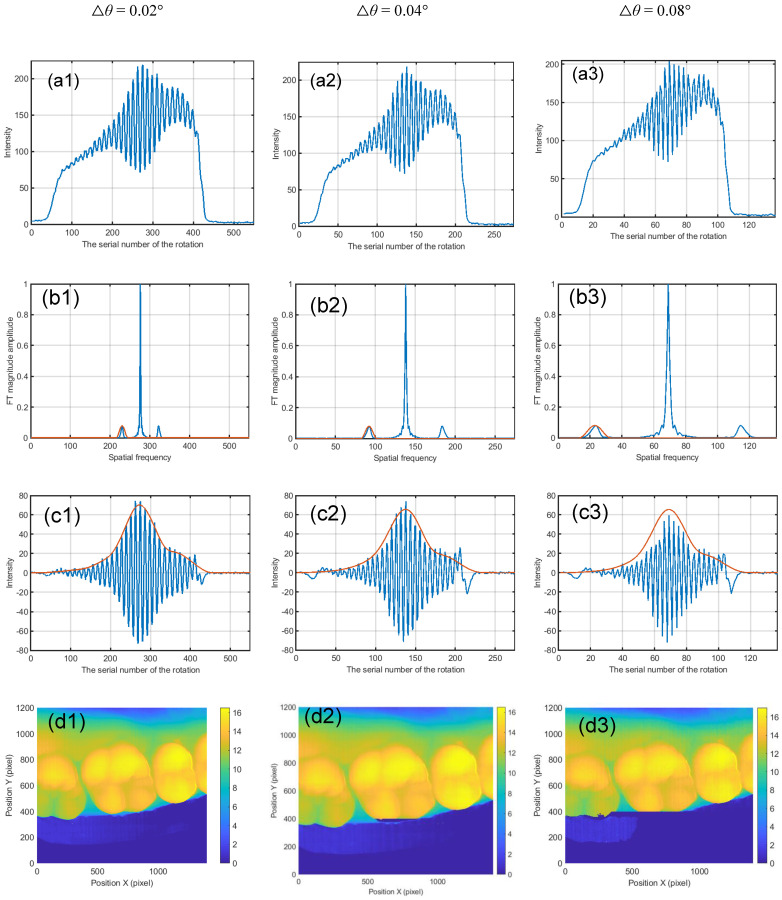

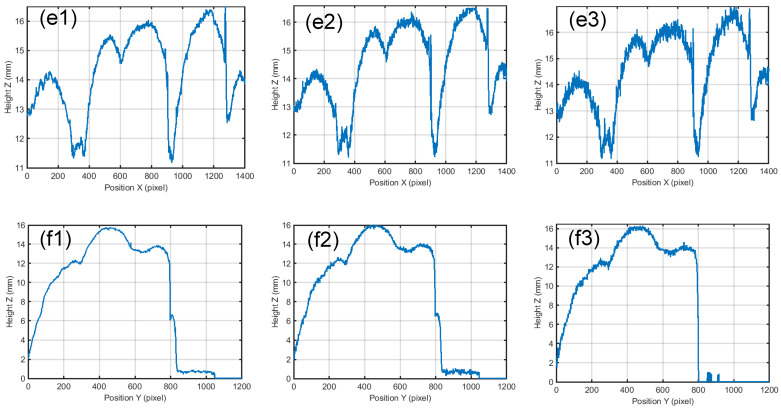
Experimental results for the measurement of a tooth model when the rotation intervals are 0.02°, 0.04° and 0.08°. (**a1**–**a3**) The intensity curve of the marker point in Figure 7a–c. (**b1**–**b3**) Magnitude of Fourier transform of signal in (**a1**–**a3**) and single-sideband filter. (**c1**–**c3**) Its intensity curve without the background and its modulation envelope. (**d1**–**d3**) The overall 3D reconstruction. (**e1**–**e3**) Cross section of row 408. (**f1**–**f3**) Longitudinal section of column 534.

To show the effect of the sampling steps, another two constructed results are calculated by selecting some images from a total of 550, i.e., it assumed that the rotation intervals are 0.04° and 0.08° separately. The experimental results are shown in Figure 8. Figure 8a1–a3 are the intensity curves of a pixel marked with a red dot in Figure 7a–c. Figure 8b1–b3 show the magnitudes of the Fourier transform of signal in Figure 8a1–a3 and single-sideband filter. It is clear that the background of the fringe intensity is not uniform. Additionally, the background intensity becomes larger at the right side. The reason for this is as follows: the left part is closer to the projecting lens, which means that the left part will be brighter. And, for any object point, its intensity curve will collect the fringe image from left to right with the mirror’s rotation. In order to see the modulation curve more clearly, the background is removed as shown in Figure 8c1–c3. The calibration range of system measurement is 20 mm, which can cover the whole tooth model. A look-up table is established between the index corresponding to the maximum modulation and physical height by calibrating the plane with an interval of 4 mm. The height of the tooth is acquired by searching the calibrated mapping stored in the computer. The reconstructed tooth model is shown in Figure 8d1–d3, Figure 8e1–e3 shows the cross sections of the column 408, and Figure 8f1–f3 shows the longitudinal section of line 534. Apparently, the experimental results are in accordance with the simulations’, and the large interval will shorten the measuring time and reduce the measuring accuracy. In the experiment, it is clear that the non-uniform illumination brings less influence while extracting the modulation curve.

Note that there are burrs at some edges of the teeth as shown in Figure 8e1–e3. It is introduced by the imaging lens and can be solved by a telecentric imaging strategy.

**Figure 9:**
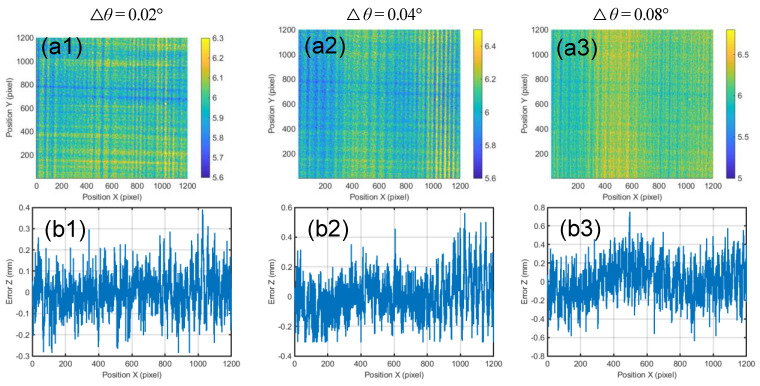

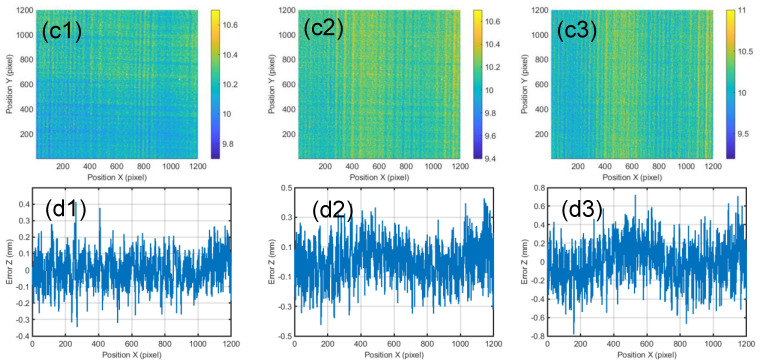
The recovery results of planes at different heights when the rotation intervals are 0.02°, 0.04° and 0.08°. (**a1**–**a3**) Reconstruction of the plane with a height of 6 mm. (**b1**–**b3**) The error distribution for the 400th row of the reconstructed plane with a height of 6 mm. (**c1**–**c3**) Reconstruction of the plane with a height of 10 mm. (**d1**–**d3**) The error distribution for the 400th row of the reconstructed plane with a height of 10 mm.

To estimate the precision of our method, we measure two reference planes at 6 and 10 mm. The reconstructed results are shown in Figure 9a1–a3 and Figure 9c1–c3. The error distributions for the 400th row of the two reconstructed planes are, respectively, shown in Figure 9b1–b3 and Figure 9d1–d3.

The mean heights of the planes and the root mean square (RMS) errors for the two planes are shown in Table 1, respectively. Obviously, the smaller sampling step will produce a smaller RMS at the same noise level. Therefore, the proposed method is expected to produce a higher accuracy, when noise level, e.g., photon noise, is reduced.

## 5. Conclusions and Discussion

In conclusion, an oblique setup of the grating is introduced to achieve uniaxial 3D measurement in this paper. Compared with the traditional uniaxial measurement techniques, the grating is set to be obliquely along instead of perpendicular to the optical axis. Therefore, the focused image plane will be tilted along the optical axis. Another special design is to introduce a mirror to reflect the projecting axis 90 degrees. Then, the FIP can be shifted by a rotation strategy instead of the classical translation movement. In other words, by rotating the mirror, the FIP will be shifted across the measured object. Therefore, for a single object point, there are two phenomena simultaneously, one is the shift of the defocusing position, the other is the phase-shift. Additionally, w.r.t the projected fringe patterns, the intensity curve of each camera pixel stems from the intensities of its neighboring fringes, except that of itself. It can be treated as a cross-correlation rather than the self-correlation.

A drawback of the proposed method is that the projecting axis could not always be parallel to the imaging axis while rotating the mirror. However, it still can be considered as keeping the merits of the uniaxial measurement while the rotation angle is not large. On the other hand, the mechanism of the rotation scanning could make the measurement system more flexible and compact. The simultaneous auto-synchronous phase-shifting can lead to a more efficient method to obtain the modulation curve and avoid a mechanical moving component to shift the grating. Moreover, the use of the physical grating can save the expensive electronic devices for digital phase-shifting. Though the current system theoretically works well, it is not with the optimal setup considering the rotation controlling and height calibration. Our future work will focus on optimizing the system to further improve the measurement accuracy.

## Figures and Tables

**Table 1:** Testing results of the two planes.

Rotation Interval △*θ*	Height = 6 mm		Height = 10 mm	
	Mean	RMS	Mean	RMS
0.02°	5.9016	0.1169	10.1104	0.1157
0.04°	5.9464	0.1607	10.0481	0.1507
0.08°	5.9545	0.2261	10.0258	0.2328
